# Anti-deception: reliable EEG-based biometrics with real-time capability from the neural response of face rapid serial visual presentation

**DOI:** 10.1186/s12938-018-0483-7

**Published:** 2018-05-03

**Authors:** Qunjian Wu, Bin Yan, Ying Zeng, Chi Zhang, Li Tong

**Affiliations:** 1 China National Digital Switching System Engineering and Technological Research Center, Zhengzhou, China; 2 Key Laboratory for NeuroInformation of Ministry of Education, School of Life Science and Technology, University of Electronic Science and Technology of China, Chengdu, China

**Keywords:** Electroencephalogram, Biometric, Face rapid serial visual presentation, Identity authentication, Anti-deception, Robustness

## Abstract

**Background:**

The electroencephalogram (EEG) signal represents a subject’s specific brain activity patterns and is considered as an ideal biometric given its superior invisibility, non-clonality, and non-coercion. In order to enhance its applicability in identity authentication, a novel EEG-based identity authentication method is proposed based on self- or non-self-face rapid serial visual presentation.

**Results:**

In contrast to previous studies that extracted EEG features from rest state or motor imagery, the designed paradigm could obtain a distinct and stable biometric trait with a lower time cost. Channel selection was applied to select specific channels for each user to enhance system portability and improve discriminability between users and imposters. Two different imposter scenarios were designed to test system security, which demonstrate the capability of anti-deception. Fifteen users and thirty imposters participated in the experiment. The mean authentication accuracy values for the two scenarios were 91.31 and 91.61%, with 6 s time cost, which illustrated the precision and real-time capability of the system. Furthermore, in order to estimate the repeatability and stability of our paradigm, another data acquisition session is conducted for each user. Using the classification models generated from the previous sessions, a mean false rejected rate of 7.27% has been achieved, which demonstrates the robustness of our paradigm.

**Conclusions:**

Experimental results reveal that the proposed paradigm and methods are effective for EEG-based identity authentication.

## Background

Identity authentication is an essential safety precaution in our daily lives, national security, public security, e-commerce, and other important areas. The rapid progress of information technology brings convenience to people but also poses great challenges to identity security. Authentication through only accounts and passwords cannot guarantee security in important places. Identity authentication based on biometric traits has recently become a hot issue because of their accuracy and portability.

Traditional biometric traits, such as faces [1], fingerprints [2], voiceprints [3], and irises [4], have a high degree of discrimination and are widely used. However, most of these traits are easy to steal and forge given their exposure to the external world. Therefore, researchers have committed to discover new internal biometric traits that cannot be remotely obtained and easily forged. Among existing biometric traits, bioelectrical signals, such as electrocardiogram (ECG) [5], electromyogram (EMG) [6], electrooculogram (EOG) [7], and electroencephalogram (EEG) [8], can satisfy the security requirement in identity authentication. EEG signals, which originate from neurons in the brain, have drawn considerable interest from researchers. EEG can be a novel biometric trait because imitating one’s mind is impossible [9] and an individual’s neural activity pattern is unique [10]. This trait can change the traditional “pass-word” into the “pass-thought.” Furthermore, external pressure will significantly influence EEG signals, thus making the EEG-based identity authentication systems capable of non-coercion [11].

Numerous EEG-based identity authentication methods have been proposed based on unique EEG features. These methods can be roughly divided into the two categories of spontaneous or evoked EEGs based on the absence or presence of a stimulus. The former includes rest eyes-open/eyes closed (REO/REC), whereas the latter involves visual evoked potentials (VEPs), mental tasks, and emotional stimuli.

In 1999, Poulos et al. developed the first identity authentication system based on EEG signals [12]. They collected the EEG data of 4 users and 75 imposters under REC conditions. Auto regressive parameters and learning vector quantization network were adopted, and the correct recognition rates of 72–84% were achieved. Palaniappan et al. constructed a dataset of VEP signals from 20 subjects [13]. The subjects focused on recognizing stimulus images from the Snodgrass and Vanderwart picture set [14]. The highest accuracy of 92.84% was obtained using the simplified fuzzy adaptive resonance theory. Sun et al. collected the EEG signals of nine subjects while they imagined moving their right or left index finger. The researchers concluded that imagining the movements of the left index finger is more appropriate for identity identification with an accuracy of 95.6% [15]. M. Abo-Zahhad et al. proposed a novel authentication system based on the fused features of EEG and EOG. The lowest verification equal error rates (EERs) were achieved using score fusion for relaxation and VEPs with EERs of 1.3 and 1.41%, respectively, in a database of 22 subjects [16]. Although these previous works obtained successful performances, the internal uniqueness of the elicited EEG signals remains unconfirmed. Moreover, most of the EEG-based authentication methods are under off-line analysis or require too much time for one-time authentication.

Evoking strong and stable individual difference is crucial in EEG-based identity authentication systems. An interesting and meaningful study was accomplished by Yeom et al. [17]. They used self- or non-self-face images as stimulus to evoke subject-specific brain activities based on neurophysiological evidence from both EEG [18] and fMRI [19]. In the field of cognitive neuroscience, an individual’s face is considered to be a representative stimulus for visual self-representation. Unlike other visual stimuli, the brain has specific regions when performing face processing, and the brain activity response to one’s own face is markedly different from the response to familiar or unfamiliar non-self-faces [20]. Thus, a unique subject-specific brain-wave pattern called visual self-representation was elicited by Yeom’s experimental paradigm. They obtained an average accuracy of 86.1% across 10 subjects using non-liner support-vector machine. However, completing one-time authentication required at least 31.5 s in their research, rendering their technique impractical. In addition, no real imposter was used to test the system’s performance.

In this paper, we propose a novel EEG-based identity authentication paradigm using self- or non-self-face images that are organized by rapid serial visual presentation (RSVP) [21]. In the RSVP paradigm, the stimulus images are presented one-by-one in a certain order and in the same position of the screen for the same presentation time. The RSVP paradigm can present a large number of stimuli in a short time and thus elicit strong event-related potentials (ERPs) [22]. The latency, amplitude, or shape of ERPs vary across subjects because of the inherent subject-to-subject variation in the neural pathways of the brain [23].

Compared with previous works, we elicited stronger subject-specific ERPs in less time through our face RSVP paradigm. Thus, the real-time capability and accuracy of the system are significantly improved. A preliminary partial version of our research was proposed in [24]. In the present study, we expanded the database of the system users and adopted a different classification method to obtain better accuracy. Two different fraud scenarios were simulated to test the system, which could demonstrate the system has the ability of anti-deception. In addition, another data acquisition session with a mean time interval of 30 days from the first acquisition is conducted for each user to evaluate the stability of our paradigm. The experiment results reveal the robustness of our system.

## Methods

### Self- or non-self-face RSVP paradigm design

#### Main framework design

**Fig. 1:**
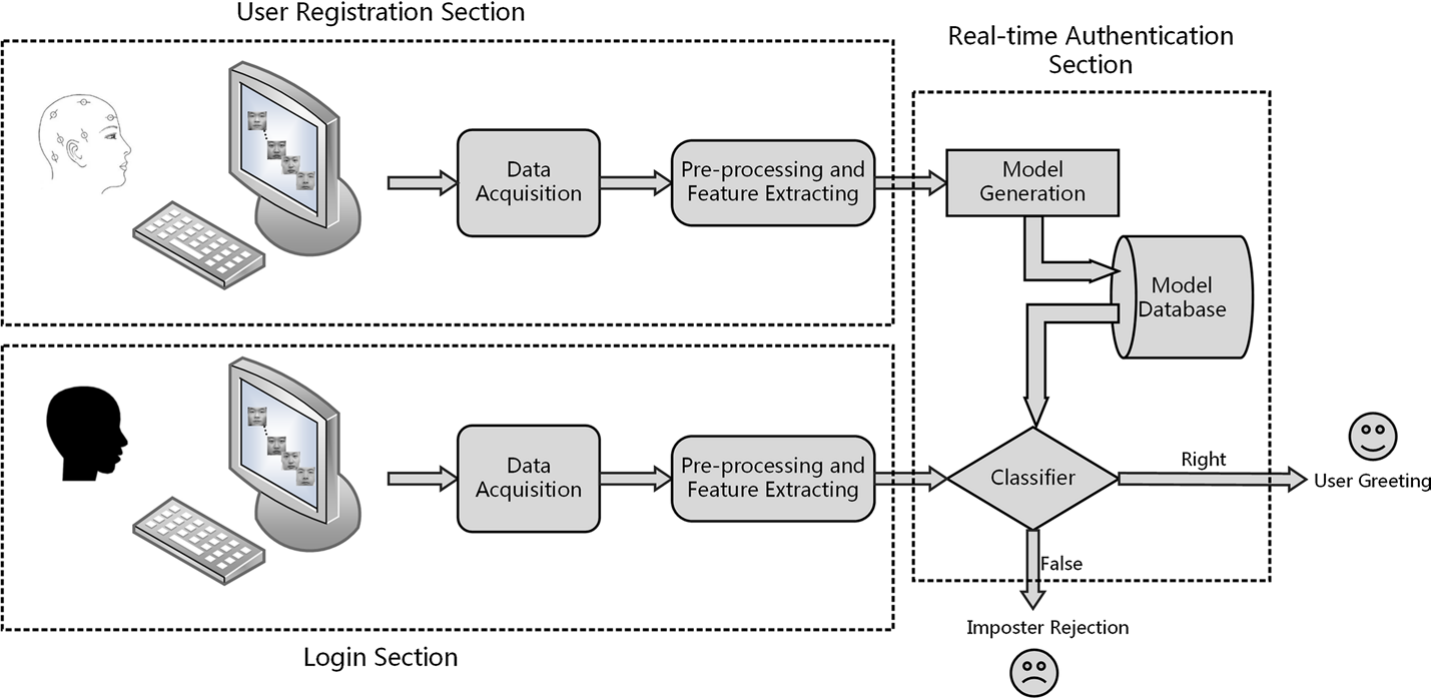
Flowchart of the authentication system design

The overall design of the EEG-based authentication system is shown in Fig. 1. During the registration section, the user is asked to focus on the face-RSVP stimulus, and the EEG signal is collected to simultaneously generate the model of the specific user. The model is stored in the database to provide data support for the classifier in the next phase. In the login section, the same stimulus is shown to the tester, and the EEG signal of the stimulus is submitted to the classifier for judgment.

#### Details of the experiment paradigm

In our experiment, the RSVP is composed of self- or non-self-face images; the self-images stand for the user’s own face, and the non-self-face images include both his/her familiar faces or unfamiliar faces. All face images present only facial information and no expression. Each image is resized to 400 × 400 pixels.

**Fig. 2:**
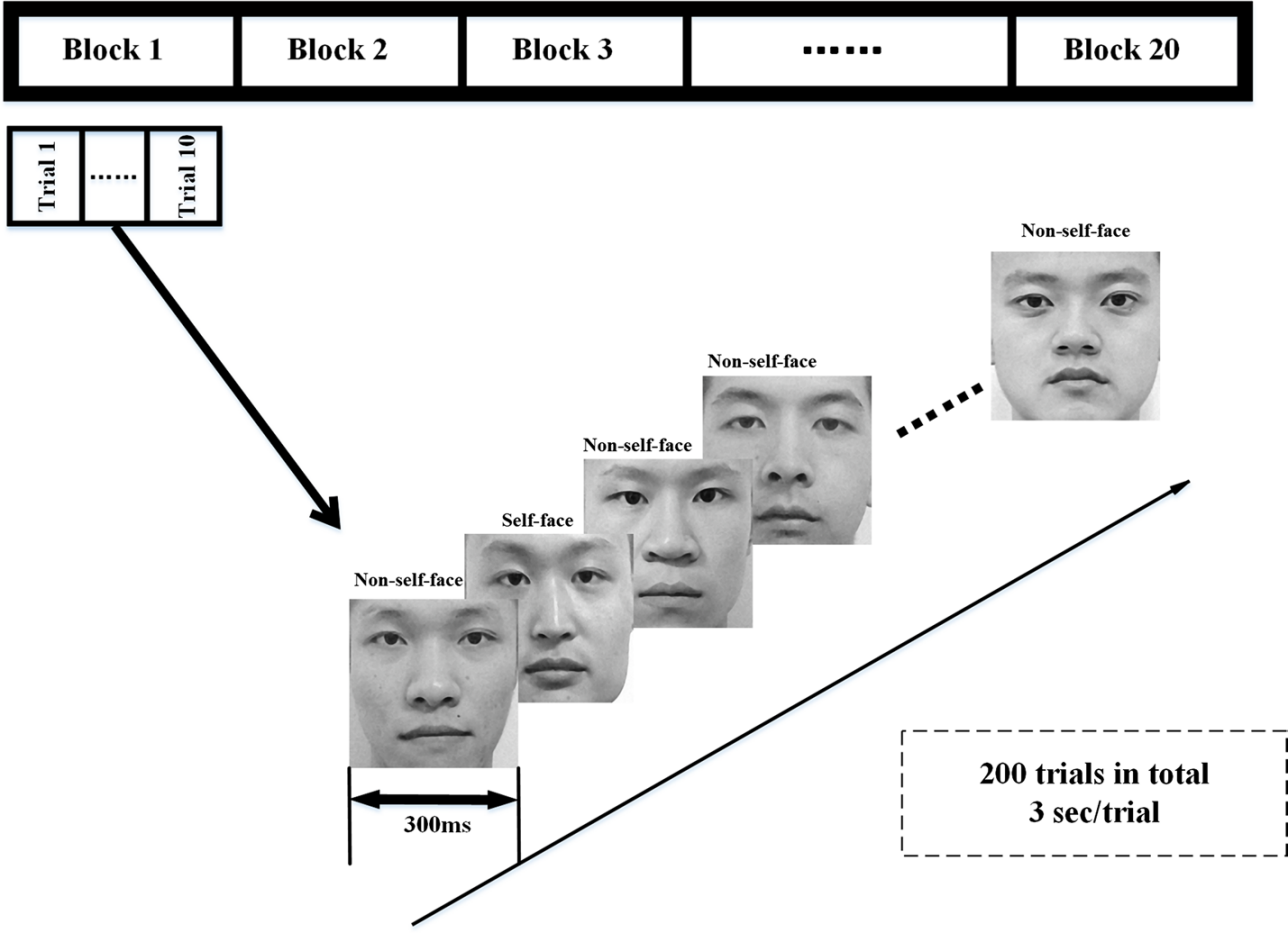
Details of the experimental stimulus

The RSVP stimulus is written in Qt 5.5.0 (a cross-platform C++ graphical user interface application development framework developed by Qt Company) and is presented at the center of the screen with a refresh rate of 60 Hz. Each RSVP trial is composed of 1 self-face image and nine non-self-face images, and the presentation time of each image is 300 ms. The presentation order of the self- or non-self-face images in each trial is randomized to avoid the effect of subject prediction on the next stimulus. The dataset consists of 20 blocks, and each block consists of 10 trials (for the trials in the same block, 10 of the face images are same but in different random order), as shown in Fig. 2. The experiment is conducted in a quiet environment. A short rest comes after 10 blocks. Each subject has 200 trials in our dataset.

### Participants

We recruited 45 subjects (15 users and 30 imposters, age range of 19–23) for the experiment. Each user has two corresponding imposters. All participants are college students, right-handed, and have normal or corrected-to-normal visual ability. None of the participants has a history of neurological disease. This study was conducted after we acquired informed consent and Ethics Committee approval of China National Digital Switching System Engineering and Technological Research Center. All of the participants have signed their written informed consent before participating and obtained a payment after completing the experiment.

### Data acquisition

The data acquisition consists of two sessions. In the session 1, the EEG signals of 15 users and 30 imposters were collected. For each user, during his specific face RSVP stimulus, we ask him to focus on his own face images and count the number of occurrences of the self-face images in his mind. For the two corresponding imposters, we simulated two different fraud scenarios. In the first scenario, the imposter does not know the user and optionally observes the face stimulus. In the second scenario, the imposter knows the user and tries to cheat the system using the same strategy of the user. Each user and his/her corresponding imposters receive the same RSVP stimulus. In the session 2, the EEG signals of each user were acquired again with the same stimulus. The mean time intervals of the session 1 and session 2 is about 30 days.

**Fig. 3:**
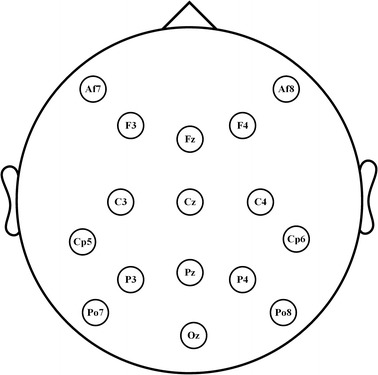
Electrode positions of the 16 channels

The EEG signals are recorded using a g.USBamp amplifier with 16 wet active electrodes. The sampling rate is 2400 Hz. As shown in Fig. 3, the 16 channels are as follows: Fz, Cz, P3, Pz, P4, Po7, Oz, Po8, C3, C4, F3, F4, Af7, Af8, Cp5, and Cp6. The raw EEG data are filtered by a low-pass Chebyshev digital filter with a passband of 40 Hz and a stopband of 49 Hz for further analysis [25]. Data are downsampled from 2400–600 Hz by averaging four consecutive samples. Finally, the data are epoched to a range of − 200 to 1000 ms with respect to stimulus onset, and the former interval data from − 200 to 0 ms are used as the baseline.

### Channel selection

**Fig. 4:**
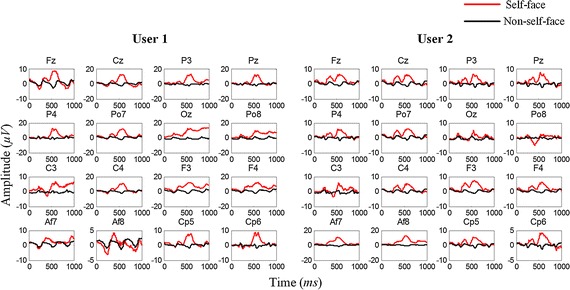
Averaged ERPs of self-face and non-self-face stimuli in two different users. A distinct difference can be seen from the latency and amplitude of the ERP between the different users

To gain a comprehensive understanding of our data, we average the ERPs elicited by self-face and non-self-face stimuli. The results show an obvious distinction in the stimuli of different categories, and the latency and amplitude of the ERP components vary in different individuals, as shown in Fig. 4.

Therefore, selecting the specific channels for each user is important. Actually, channel selection is an important strategy in brain computer interface (BCI), which can not only improve the systems’ practicability, but also enhance the stability. For instance, Yin et al. proposed a channel selection method using jumpwise regression (a stepwise regression-inspired algorithm) in a P300 BCI [26]. They selected 8 channels from 32 channels and gain a satisfying result. In this paper, our selection method is based on the algorithm proposed by Yeom et al. [17]. First, we calculate the pointwise biserial correlation coefficient (referred to as the p value in the following discussion) for each channel. The p value is a special form of the Pearson product-moment correlation coefficient and is defined as follows:

1 $$P_{i} (n) = \frac{{\sqrt {N_{1} N_{2} } }}{{N_{1} + N_{2} }} \cdot \frac{{M_{i}^{SF} (n) - M_{i}^{NSF} (n)}}{S(n)}$$

where *i* denotes the number of channels, namely, *i *= 1, 2… 16; and *n* represents the sample point, namely, *n* = 1, 2… 600. *N*_1_ and *N*_2_ are the total numbers of trials of the self-face and non-self-face stimuli, respectively. *M*_*i*_^*SF*^(*n*) and *M*_*i*_^*NSF*^(*n*) are the mean values of all trials in both classes on the sample point *n*. *S*(*n*) denotes the standard deviation of all trials of both self-face and non-self-face stimuli. *P*_*i*_(*n*) increases when the EEG signals are further apart when facing the two different stimuli or when the variance is smaller. The channels with a high p value are the representative channels. Therefore, we calculate the sum of each channel’s p value and sort them in a descending order. The channels with a p value sum in the top 6 are finally selected.

### Classification with hierarchical discriminant component analysis (HDCA)

The ERPs always contain a certain degree of external noise components, and their amplitude and latency may vary a lot because of the status of users. Thus, we apply HDCA to classify the specific ERPs evoked by the face RSVP, which extracts both spatial and temporal features of the ERPs [27, 28]. The detail of the algorithm is as follows.

#### Spatial features extraction

First, each channel of the EEG signals are divided into N segments on average by the given time window. Second, using the Fisher linear discriminant analysis, the weight of each channel is calculated in each time window to maximize the difference between the target and non-target classes. Finally, the multichannel EEG signals are compressed into a single channel signal, namely,

2 $$y_{n} = \sum\limits_{i} {w_{n,i} x_{i,n} } \,\, i = 1,2,3 \ldots 6$$

where i and n denote the number of channels and EEG segments, respectively; *x*_*i*,*n*_ and *w*_*n*,*i*_ represent the *i*-th channel EEG signal in *n*-th segment and its weights; and *y*_*n*_ is the desirable single channel EEG signal.

#### Temporal feature extraction

First, the segment signals of the *y*_*n*_ in each EEG are averaged to obtain a dimension signal, namely,

$$y_{k} ,k = 1,2,3 \ldots N.$$

Then, the weights of *y*_*k*_ are calculated to make the target score higher than the non-target score by using the logistic regression method, namely,

3 $$Y_{S} = \sum\limits_{k} {v_{k} y_{k} } .$$

## Results

### Average ERPs analysis

**Fig. 5:**
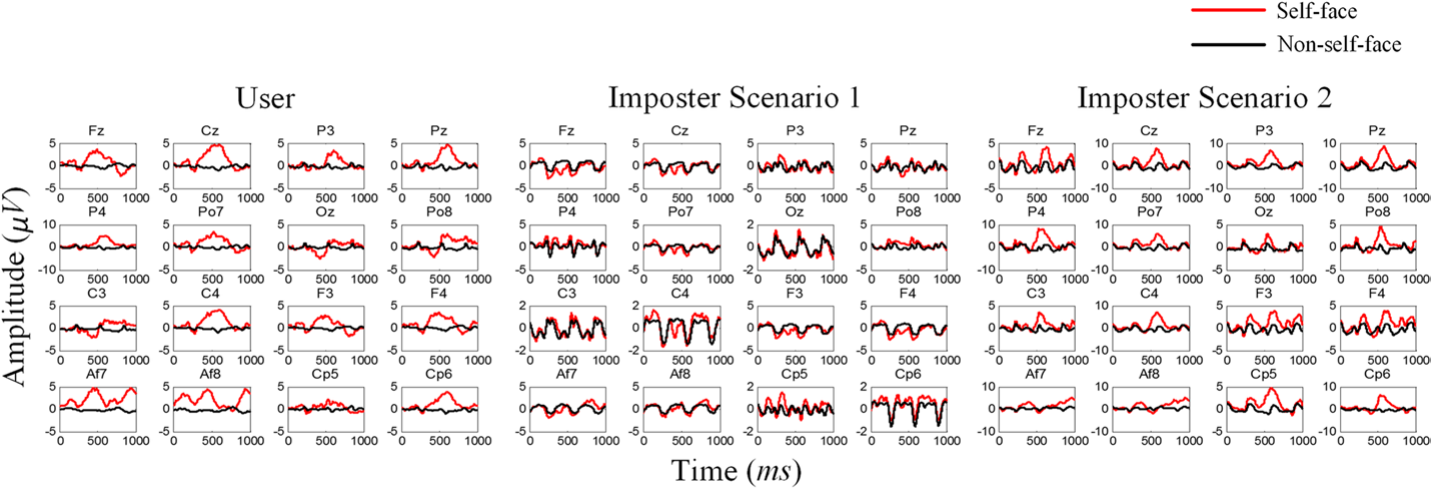
Average ERPs evoked by the self-face (red line) and non-self-face (black line) images. Note that the user and his/her corresponding imposters have same RSVP stimuli. For the user, an obvious difference is observed between the ERPs evoked by the self-face and non-self-face images. In imposter scenario 1, no apparent difference is observed between the two kinds of ERPs because observing the stimuli is optional for this person. In imposter scenario 2, although a certain difference is observed between the two kinds of ERPs, the amplitude, shape, and latency are distinctly different from those for the user

To validate the effectiveness of the designed experimental paradigm, we analyze the average ERPs in the first stage. The average ERPs of a real user and two corresponding imposters in different scenarios are shown in Fig. 5. N250, which is a main ERP component related to face stimulus according to previous EEG evidence, can be observed clearly in both user and imposters [29]. For the user, an obvious difference is observed between the ERPs evoked by the self-face and non-self-face images, and the difference is specific to an individual. For imposter 1, no apparent difference is observed between the two kinds of ERPs because observing the stimuli is optional for this person. For imposter 2, although a certain difference is observed between the two kinds of ERPs, the amplitude, shape, and latency are distinctly different from those for the user. Furthermore, the channel location of the difference in the imposter varies from that in the user, which justifies channel selection.

**Fig. 6:**
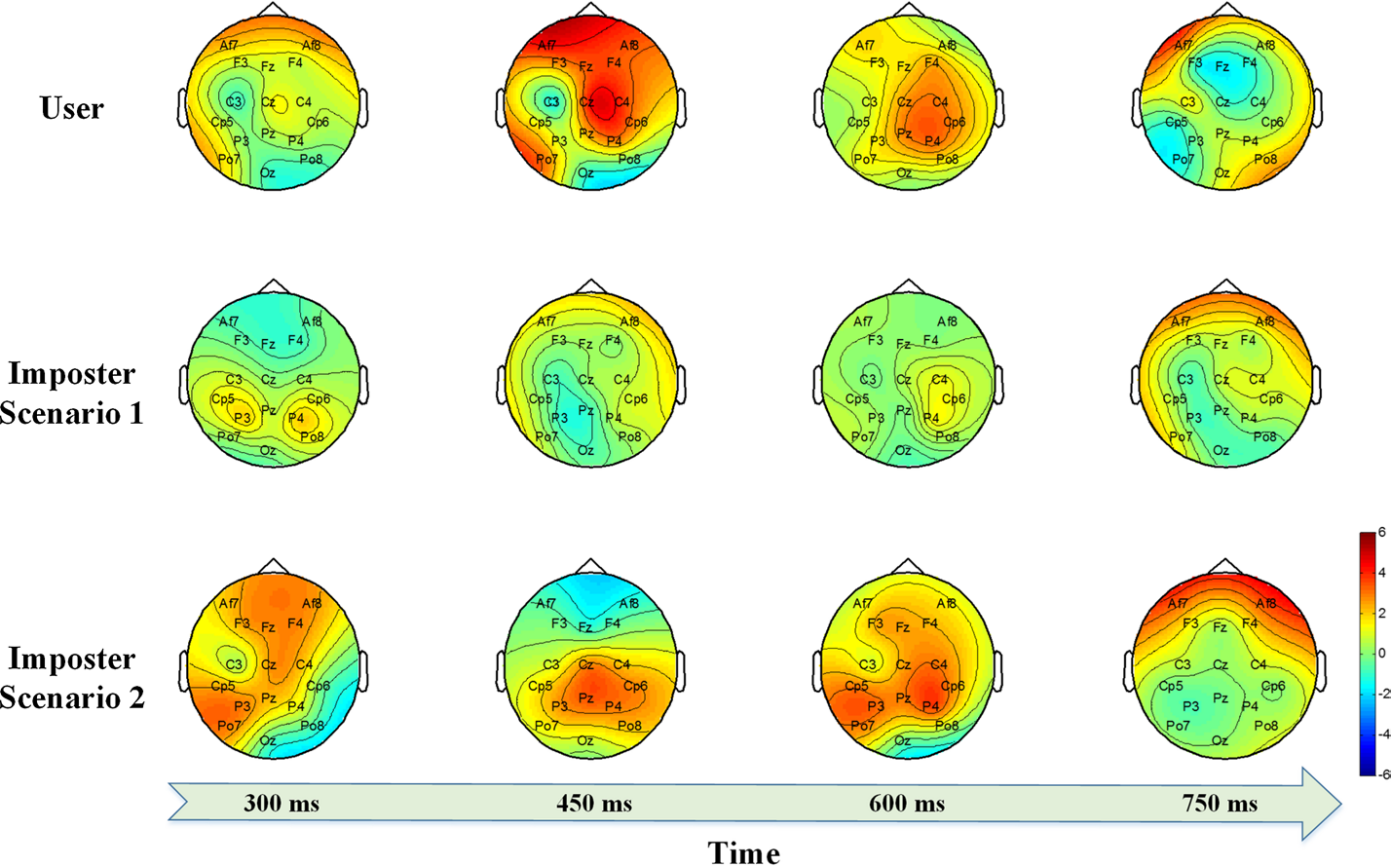
The ERP topographical maps. The brain activation intensity and region is distinctly different between the user and two imposters

The individual differences in the ERP topographical maps of the user and the two imposters are clearly observable in Fig. 6. In summary, individual-specific ERP characteristics are evoked by the self- or non-self-face RSVP paradigms and are difficult to be forged by the imposter.

### Classification result analysis

#### Classification scene settings

The classification tests are composed of two sections. In section 1, we conduct a 10-fold cross-validation for each user by the EEG signals (both the user’ data and its corresponding two imposters’ data) collected in session 1. The classification accuracy (ACC), false acceptance rate (FAR), and false rejection rate (FRR) are used to evaluate the performance of the system of each user, which are defined as follow:

4 $$ACC = \frac{number \, of \, correctly \, authenticated \, samples}{total \, number \, of \, test \, samlpes}$$

5 $$FAR = \frac{number \, of \, falsely \, accepted \, samples}{total \, number \, of \, imposter \, test \, samlpes}$$

6 $$FRR = \frac{number \, of \, falsely \, rejected \, samples}{total \, number \, of \, user \, test \, samlpes}$$

Then, a classification model could be generated for each user in this section. In section 2, each user’s EEG signals, which are acquired in session 2, are classified using the classification model generated from section 1. Thus, FRR is adopted to evaluate the performance in section 2.

In classification stage, we first average two adjacent single trials to obtain more stable and less noisy EEG signals. Thus, completing a one-time authentication takes 6 s, which is acceptable in practical application. In section 1, there are 100 average trials signals for each user and imposter. In section 2, there are 100 average trials signals for each user.

#### Classification results in section 1

In this section, we then implement a 10-time, tenfold cross-validation to obtain the mean accuracy per user, where we randomly select 90 trials for training and use the remaining 10 trials for verification.

The classification accuracy, false acceptance rate (FAR), and false rejection rate (FRR) in two different scenarios are shown in Table 1. It can be seen from the results that the paradigm we design has a desirable performance in EEG-based identity authentication under both of the scenarios. In the imposter scenario 1, the system gets a mean accuracy of 91.31%, FAR of 9.53%, and FRR of 7.86%; In the imposter scenario 2, the system gets a mean accuracy of 91.61%, FAR of 8.93%, and FRR of 7.85%. As a result, it can be concluded that even though the imposter tries to imitate the user’s strategy, it is hard for the imposter to be accepted in the system.

**Table 1:** Performance of the EEG-based identity authentication system in section 1

Users	ACC (%)		FAR (%)		FRR (%)	
	Scenario 1	Scenario 2	Scenario 1	Scenario 2	Scenario 1	Scenario 2
1	88.9	90.9	14.4	14.3	7.8	4.0
2	91.6	98.1	8.5	2.3	8.4	1.5
3	89.0	88.0	14.0	12.5	8.1	11.5
4	95.7	94.7	6.0	7.9	2.7	2.7
5	92.9	88.4	8.2	10.4	6.0	12.8
6	95.3	90.5	5.6	9.3	3.8	9.8
7	88.4	90.9	9.4	11.1	13.9	7.1
8	91.1	98.1	11.1	3.0	6.7	0.8
9	88.4	93.0	8.8	5.6	14.4	8.5
10	90.4	87.3	10.4	15.8	8.9	9.7
11	96.6	91.4	2.2	8.5	4.6	8.7
12	90.7	89.2	11.9	7.3	6.8	14.3
13	91.1	92.2	8.4	6.6	9.4	9.1
14	88.6	92.0	17.6	11.4	5.3	4.7
15	91.3	89.8	6.4	7.9	11.1	12.5
Single-mean (std)	91.31 (2.71)	91.61 (3.27)	9.53 (3.91)	8.93 (3.80)	7.86 (3.38)	7.85 (4.25)
Ensemble-mean (std)	91.46 (2.96)		9.23 (3.80)		7.85 (3.77)	

#### Classification results in section 2

In order to test the system stability, a second data acquisition session was conducted for each user. The average time interval between the first session and second session is about 30 days. In this section, the EEG signals, which are acquired in session 2, are classified using the classification model generated from section 1. The performance of this section is shown in Table 2. A mean FRR of 7.24% can be achieved. The test results illustrate the stability of our visual evoked paradigm, which is essential for an EEG-based person authentication system.

**Table 2:** The false rejected rate of each user in section 2

User	FRR (%)
1	6
2	10
3	3
4	12
5	11
6	8
7	9
8	8
9	9
10	14
11	0
12	5
13	10
14	0
15	4
Mean (std)	7.27 (4.18)

## Discussion

We propose a novel EEG-based identity authentication algorithm based on self-or non-self-face RSVP. We reveal that the specific face RSVP stimulus elicit distinct biometrics in each user. These distinct biometrics can achieve a satisfactory authentication accuracy in real-time conditions. Below, we provide a detailed discussion of our results for a more complete exposition of the performance of our algorithm.

### Comparison with existing EEG-based identity authentication systems

An increasing number of studies have been recently conducted to improve the performance of EEG-based identity authentication systems. A comparison of our method with previous related works is provided in Table 3. The superiority of our proposed method can be seen from the performance comparison. For example, Yeom et al. [17] achieved a mean accuracy of 86.1%, FAR of 13.9%, and a FRR of 13.9% in 10 users. In our proposed method with a larger database, the mean accuracy of 91.46% is higher, whereas the FAR of 9.23% and FRR of 7.85% are lower. To test our system, we designed two different real imposter scenarios, which were not considered by the previous studies. The stability tests for each user reveal the robustness of our paradigm. Furthermore, completing one-time authentication costs only 6 s in our system, which shows better real-time performance than previous studies.

**Table 3:** Performance comparison of the previous works

Authors	Stimulus type	Cost of one-time authentication (s)	Imposter scenarios	Stability test	Accuracy (%)	FAR (%)	FRR (%)
Armstrong et al. [30]	Text reading	NA	None	Yes	89	NA	NA
Yeom et al. [17]	Self-or non-self-face images	31.5–41	None	None	86.1	13.9	13.9
Marcel et al. [31]	Motor imagery	15	None	None	80.7	14.4	24.3
Miyamoto et al. [32]	Resting state	60	None	None	79.0	21.0	21.0
Zhendong Mu et al. [33]	Self- and non-self-photos	6.5	None	None	87.3	5.5	5.6
Proposed method	Face RSVP	6	2 scenarios	Yes	91.46	9.23	7.85

### Necessity of the channel selection

**Fig. 7:**
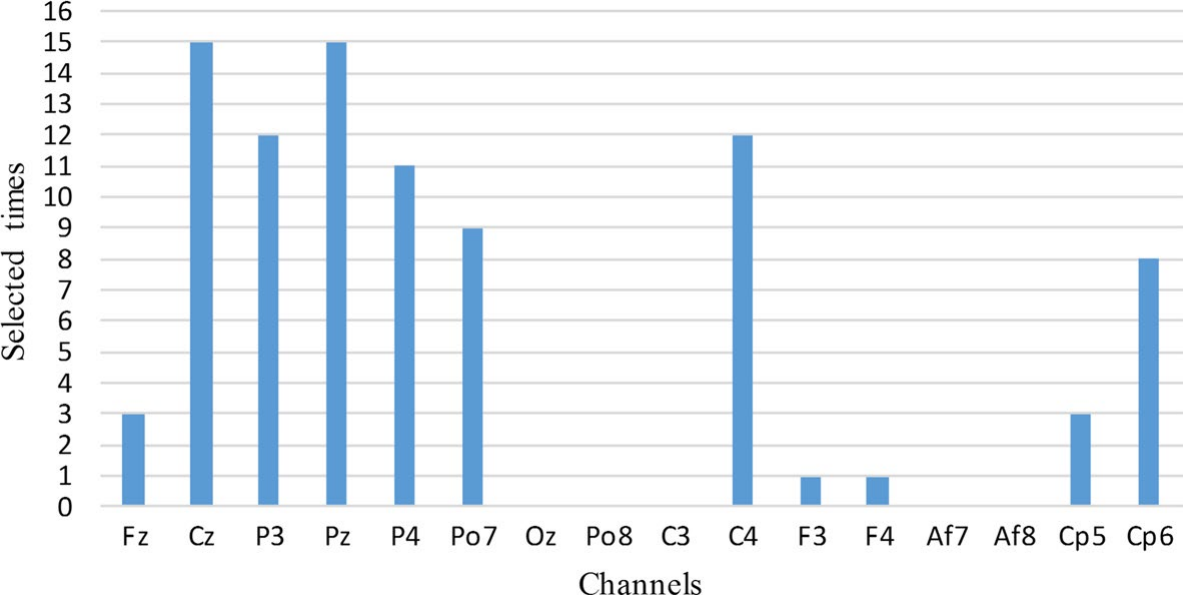
The selected times of each channels. The most relevant electrodes of our stimulus are “Cz” and “Pz” because they are adopted by each user. The selected times of “P3”, “P4”, and “C4” are also relatively high. The selected channels are mainly distributed in the central and parietal areas, which might be are mainly responsible for the self-or non-self-face RSVP

Channel selection serves two purposes. The first is to enhance practicality. The EEG signal is a multi-channel signal. Thus, the portability of the system can be improved by selecting channels with representative information and reducing the number of channels. The second is to enhance recognition rate. As shown in Fig. 6, the activation areas of the brain are significantly different between the user and the imposter. Therefore, it’s important to establish the specific classifier for the user using the specific channels of the user, which can make the system resistant to forgery. In this study, we select six specific channels for each user. The selected channels are detailed in Table 4. Each user has their specific channel combination. We calculate the selected times of each channel as shown in Fig. 7. The most relevant electrodes of our stimulus are “Cz” and “Pz” because they are adopted by each user. The selected times of “P3”, “P4”, and “C4” are also relatively high. As a result, we found that the selected channels are mainly distributed in the central and parietal areas. Thus, these areas are mainly responsible for the self-or non-self-face RSVP.

**Table 4:** The selected channels for each user

User	The selected top 6 channels					
	1	2	3	4	5	6
1	Cz	Pz	Po7	P3	C4	P4
2	Pz	P3	P4	Cz	Cp6	Po7
3	Cp6	Pz	P4	P3	Cz	C4
4	Cz	Pz	P4	P3	Po7	C4
5	P4	Pz	C4	Cz	Cp6	P3
6	C4	Cz	P4	Pz	Cp6	P3
7	P4	Pz	C4	Cz	Cp6	P3
8	Cz	C4	Pz	Cp6	Po7	P4
9	Pz	Cz	P4	P3	Po7	C4
10	Cz	C4	P3	Pz	Po7	Cp5
11	Cz	Pz	Po7	C4	P3	Fz
12	Cz	Fz	C4	Po7	Pz	Cp6
13	Cz	Po7	Pz	F4	Fz	F3
14	Pz	P4	Cz	P3	C4	Cp5
15	Pz	P4	P3	Cp6	Cp5	Cz

### Simulation of imposter scenarios

**Fig. 8:**
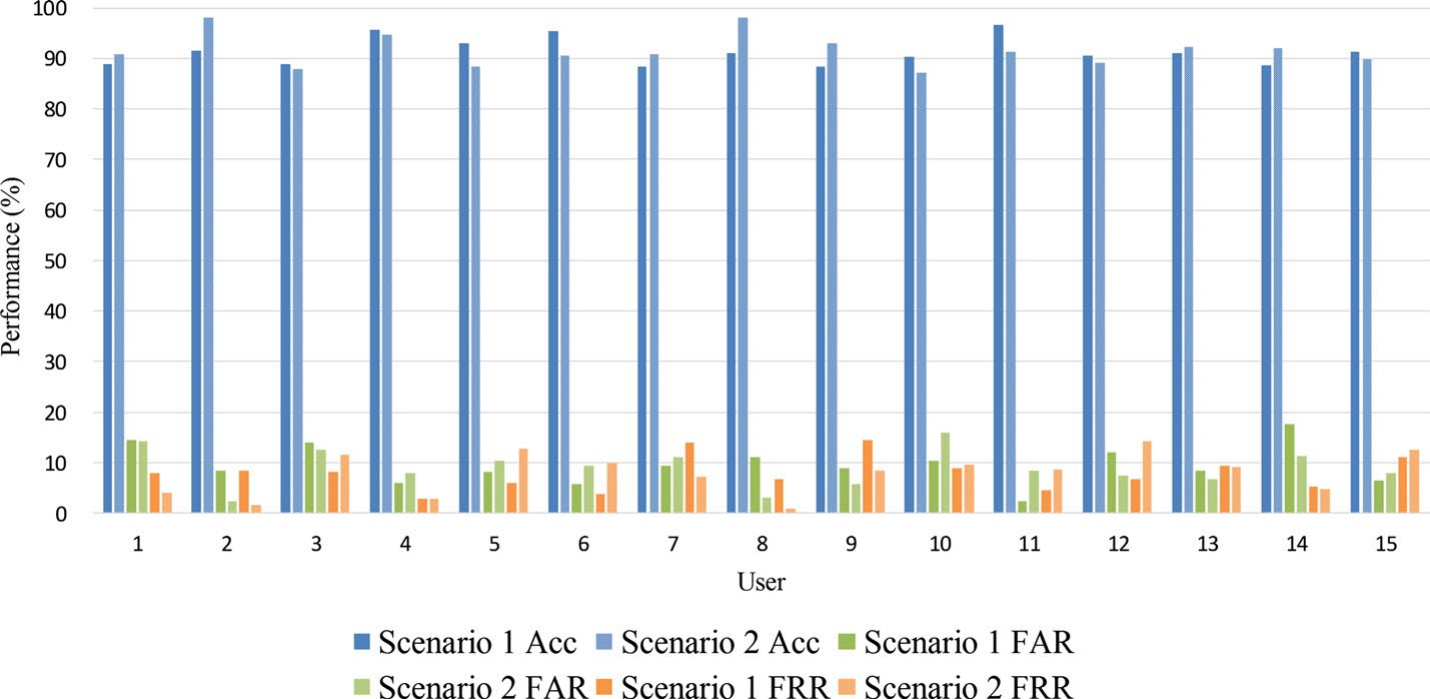
The contrast of the two scenarios results. The paradigm gets a good performance in both two scenarios, which indicates our system has the ability of anti-deception

Two scenarios are designed to simulate fraud behavior in practical applications. In scenario 1, the imposter just observes the face stimulus optionally. However, in scenario 2, the imposter focuses on the user’s face image, and performs the same strategy of the user. As shown in Table 1, we obtained a satisfactory performance in both two imposter scenarios because although the imposter makes every effort to imitate the user’s behavior, he cannot imitate the brain activity of the user. Figure 8 vividly shows the contrast between the results from the two scenarios. Thus, our system has the ability to prevent cheating.

### Permanence of the face-RSVP-evoked EEG biometric

**Fig. 9:**
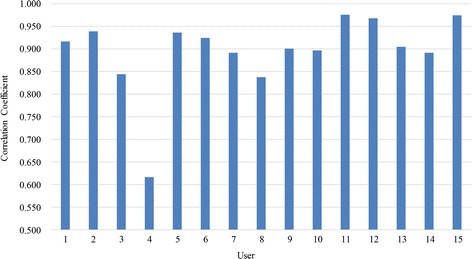
The correlation coefficients of the selected 6 channels EEG signals between the two acquisition sessions

Maintaining permanency over a long period of time is a basic requirement for the practical applications of a biometric trait. Armstrong et al. found the stable ERP biometric elicited by the text reading in the time interval of 6 months [30]. In our work, we found that the EEG signals evoked by our face RSVP paradigm are relatively stable over 30 days. The correlation coefficients of the selected 6 channels EEG signals between the two acquisition sessions for each user are shown in Fig. 9. A mean correlation coefficient of 0.894 is achieved. The correlation coefficient for all other users can reach above 0.84 except the user 4. In our future work, it’s meaningful and necessary to repeat the experiment after a few months or even a few years to explore the permanence of the evoked EEG biometric.

### Future directions

Our method reveals the potential of using EEG as an ideal biometric. However, there are something we need to consider in the future work.

On the one hand, our experiments are conducted in the normal state of subjects. In the future research, the subject features, such as gender, age, fatigue, mood, and so on, should be recorded and analyzed. Furthermore, the external environment factors, such as light interference and electromagnetic interference, should also be tested.

On the other hand, most data acquisition of EEG is inconvenient at the current stage. We have to place many electrodes on the scalp and use conductive gel to reduce skin impedance. Thus, channel selection is adopted in this paper, which can not only improve the accuracy of the system, but also ameliorate the portability. Moreover, with the development of technology, wireless EEG devices with dry electrodes have been produced. Although the signal quality of these devices is poor, this is the first step for practical application.

## Conclusion

In recent years, many studies have been conducted on the use of EEG signals given their potential as reliable biometric traits and satisfactory performance in forgery prevention. We proposed a novel EEG-based identity authentication method based on self-or non-self-face RSVP. Using our paradigm, a distinct and stable biometric trait is elicited with a lower time cost of 6 s. Channel selection is performed to enhance system portability and improve the identification of user and imposter. We also found that the central and parietal areas might be responsible for the self-or non-self-face RSVP stimulus. In the classification stage, we adopt the HDCA algorithm, an effective method for the recognition of RSVP-evoked EEG signals. Two different imposter scenarios are designed to test the paradigm, which exhibit the capability to prevent fraud. The stability tests for each user in two independent session demonstrate the robustness of our paradigm. In future work, we will repeat the experiment after a few months to further explore system stability. Commercial portable EEG acquisition equipment, such as the Emotiv EPOC headset, will be used to improve system practicability.

## Abbreviations

EEG: electroencephalogram
ECG: electrocardiogram
EMG: electromyogram
EOG: electrooculogram
REO: rest eyes-open
REC: rest eyes-closed
VEPs: visual evoked potentials
EERs: equal error rates
RSVP: rapid serial visual presentation
ERPs: event-related potentials
BCI: brain computer interface
HDCA: hierarchical discriminant component analysis
FAR: false acceptance rate
FRR: false rejection rate
